# Clinical Significance of *cfiA* Positivity Detected by Matrix-Assisted Laser Desorption/Ionization Time-of-Flight Mass Spectrometry in *Bacteroides fragilis* Infections

**DOI:** 10.3390/microorganisms14010168

**Published:** 2026-01-12

**Authors:** Wing-Man Chik, Lam-Kwong Lee, Jason Chi-Ka Cheng, Suk-Han Yuen, Rocky Shum, Gilman Kit-Hang Siu, Sandy Ka-Yee Chau

**Affiliations:** 1 Department of Pathology, United Christian Hospital, Hong Kong SAR, China; cck936@ha.org.hk (J.C.-K.C.); chauky@ha.org.hk (S.K.-Y.C.); 2 Department of Health Technology and Informatics, Faculty of Health and Social Science, The Hong Kong Polytechnic University, Hong Kong SAR, China; lamkwongeddie.lee@connect.polyu.hk (L.-K.L.); gilman.siu@polyu.edu.hk (G.K.-H.S.); 3 Department of Pathology, Tseung Kwan O Hospital, Hong Kong SAR, China; ysh989@ha.org.hk (S.-H.Y.); r.shum@ha.org.hk (R.S.)

**Keywords:** *Bacteroides fragilis*, *cfiA*, MALDI-TOF MS, carbapenem resistance, ST157, insertion sequence, *bla*_OXA-347_, *bla*_MUN_, MLST, Hong Kong

## Abstract

The MALDI-TOF MS Bruker Biotyper MBT subtyping IVD module enables the early detection of *cfiA*-positive *Bacteroides fragilis* (*cfiA*+ BF) during bacterial identification. However, the relationship between genetic positivity, phenotypic resistance, and clinical outcomes has not been fully elucidated. This retrospective study analyzed *B. fragilis* isolates from three Hong Kong hospitals between 2021 and 2025 to examine their prevalence and the clinical utility of MALDI-TOF MS in rapid *cfiA* detection. Antibiotic susceptibility testing, *cfiA* gene detection using MALDI-TOF MS, and Oxford Nanopore sequencing were performed. Medical records were reviewed, and univariate analyses and multivariate logistic regression were used to identify factors associated with *cfiA* positivity and 30-day all-cause mortality. Overall, *B. fragilis* exhibited a high rate of antibiotic resistance. Concomitant resistance to carbapenems and metronidazole was identified in three isolates. Among the 166 isolates, 40 (24.1%) were *cfiA*-positive. *cfiA* detection by MALDI-TOF MS showed 100% concordance with the gene sequencing results and correlated strongly with phenotypic carbapenem resistance (Φ = 0.82, *p* < 0.001 for meropenem; Φ = 0.70, *p* < 0.001 for ertapenem; Φ = 0.63, *p* < 0.001 for imipenem). Phylogenetic analysis revealed two distinct clusters corresponding to *cfiA* status, each exhibiting genetic diversity based on multi-locus sequence typing (MLST). The *cfiA*+ BF isolates demonstrated high-level phenotypic carbapenem resistance in the presence of upstream insertion sequences. The predominant sequence type (ST) among *cfiA*+ BF isolates was ST157, and 70% of ST157 isolates harbored IS*1187* in the upstream region of *cfiA*. Gene sequencing also identified other emerging beta-lactamase genes *bla*_OXA-347_ and *bla*_MUN_. The 30-day all-cause mortality following *B. fragilis* infection was 13.3%, with independent predictors including a high Charlson Comorbidity Index (OR = 1.30; *p* = 0.02) and the absence of early source control (OR = 4.84; *p* = 0.03). This study highlights the widespread occurrence of *cfiA*+ BF in Hong Kong and the clinical significance of rapid *cfiA* detection. Continuous surveillance is essential to monitor the ongoing threat of antibiotic resistance in *B. fragilis*.

## 1. Introduction

*Bacteroides fragilis* is a gram-negative anaerobic bacterium and a key constituent of the colonic microbiota. It is an opportunistic pathogen capable of causing a variety of human infections, including bacteremia, and intra-abdominal, skin, and soft tissue infections. The global emergence of antibiotic resistance among *B. fragilis* strains has posed a significant threat to public health [1,2,3,4]. Infections caused by resistant *B. fragilis* have been associated with an increased risk of treatment failure and adverse clinical outcomes [5,6].

Antibiotic resistance in *B. fragilis* is mediated by multiple genes, among which *cfiA* is regarded as the predominant carbapenemase gene. Molecular studies have shown that *cfiA*-negative (*cfiA*− BF) and *cfiA*-positive (*cfiA*+ BF) *B. fragilis* strains belong to two genotypically distinct divisions, I and II, respectively [7,8], which differ in their geographic distribution and pathogenic potential [9]. Most division I isolates carry the *cepA* gene, which encodes a class A serine beta-lactamase conferring resistance to penicillins and cephalosporins. On the other hand, division II isolates possess the *cfiA* gene, which encodes a class B metallo-beta-lactamase that mediates resistance to carbapenems and other beta-lactam antibiotics. Notably, not all *cfiA*+ BF isolates exhibit phenotypic resistance because the gene may only be weakly expressed in the absence of an upstream mobile element [10].

Routine antibiotic susceptibility testing of anaerobes is uncommon in many clinical laboratories because it is labor-intensive and time-consuming. Phenotypic susceptibility testing usually takes several days to complete, and many genotypic methods require specialized technical expertise [11]. Consequently, antibiotic treatment is typically empirical, guided by expected susceptibility patterns and local resistance epidemiology. In the era of rising antibiotic resistance, there is a growing need for a rapid, accurate and cost-effective susceptibility testing method.

In recent years, the new MBT subtyping IVD module has been introduced for the matrix-assisted laser desorption/ionization time-of-flight mass spectrometry (MALDI-TOF MS) Bruker Biotyper (Bruker Daltonics, Bremen, Germany), enabling rapid differentiation between *cfiA*+ BF and *cfiA*− BF at the time of bacterial identification. Previous studies have confirmed its diagnostic accuracy, but the relationship between genetic positivity, phenotypic resistance and clinical outcomes has not been fully elucidated [12,13,14,15]. In Hong Kong, where the prevalence of *cfiA*+ BF is high [16], it is particularly important to investigate how this module may be useful in clinical practice.

The aim of this study was to characterize the phenotypic and genotypic resistance patterns of *B. fragilis* from three hospitals in Hong Kong. Additionally, this study evaluated the risk factors and clinical outcomes associated with *cfiA*+ BF infections. These findings may offer insights into laboratory diagnosis, antimicrobial stewardship and clinical decision-making.

## 2. Materials and Methods

### 2.1. Patient Identification and Isolate Collection

This retrospective observational study was conducted across three non-teaching hospitals (United Christian Hospital, Haven of Hope Hospital and Tseung Kwan O Hospital) within the Kowloon East Cluster in Hong Kong from 2021 to 2025. Both United Christian Hospital and Tseung Kwan O Hospital are acute-care hospitals, while Haven of Hope Hospital is an extended-care hospital. As of 31 March 2024, the three hospitals serve a population of approximately 1,171,600, with a total of 3010 beds. Cases with *B. fragilis* infections were identified through the Laboratory Information System. Due to variations in laboratory practice pertaining to strain preservation, isolates from all specimen types were retrieved from United Christian Hospital over a 14-month period (November 2023 to January 2025), whereas only blood culture isolates were retrieved from Tseung Kwan O Hospital and Haven of Hope Hospital over a 4-year period (January 2021 to January 2025). All isolates were stored at −70 °C before testing. If multiple isolates were obtained from the same patient during the study period, only the first isolate was included, regardless of the specimen type.

### 2.2. Isolate Identification

*B. fragilis* isolates were re-cultured on blood agar and incubated at 37 °C in the Concept Anaerobic Workstation (Baker Ruskinn, Bridgend, UK). These were identified down to the species level with identification scores ≥ 2.0 using a MALDI-TOF MS Bruker Biotyper equipped with the MBT IVD Library revision J (Bruker Daltonics, Bremen, Germany) according to the manufacturer’s instructions. The MBT subtyping IVD module simultaneously reported the *cfiA* status as a categorical output (“*cfiA*-positive” or “*cfiA*-negative”) at the time of bacterial identification. The *cfiA* subtyping result was not displayed if the internal threshold values of the algorithm were not met.

### 2.3. Antibiotic Susceptibility Testing

Antibiotic susceptibility testing was performed using the disk diffusion method based on the European Committee for Antimicrobial Susceptibility Testing (EUCAST) guidelines [17]. *B. fragilis* isolates were tested against the following antibiotic disks (Oxoid Ltd., Basingstoke, UK): ampicillin–sulbactam (10 µg/10 µg), amoxicillin–clavulanic acid (2 µg/1 µg), piperacillin–tazobactam (30 µg/6 µg), ertapenem (10 µg), imipenem (10 µg), meropenem (10 µg), metronidazole (5 µg), and clindamycin (2 µg). Bacterial suspensions adjusted to 1.0 McFarland standard were incubated with antibiotic disks on fastidious anaerobe agar supplemented with 5% defibrinated horse blood in the same anaerobic workstation for 16 to 20 h. *B. fragilis* ATCC 25285 and *Clostridium perfringens* ATCC 13124 were used for quality control. The antibiotic susceptibility was interpreted according to the EUCAST breakpoint guidelines version 15.0, which was valid from 1 January 2025 [18].

### 2.4. Genomic Sequencing

All *B. fragilis* isolates were subjected to Oxford Nanopore sequencing. Bacterial DNA was extracted using the QIAamp BiOstic Bacteremia DNA Kit (Qiagen, Hilden, Germany) following the manufacturer’s protocol. Sequencing libraries were prepared using the Nanopore-only Microbial Isolate Sequencing Solution (NO-MISS) protocol for cell cultures with the Rapid Barcoding Kit 96 V14 (SQK-RBK 114.96; Oxford Nanopore Technologies, Oxford, UK). The libraries were sequenced using the R10.4.1 flow cells on the GridION MK1 platform for 72 h. Basecalling was performed using Dorado Basecall Server v7.8.3 in super-accurate (SUP) mode and reads with a Phred quality score (Q score) less than 10 were filtered out. Sequencing yield and quality were assessed with NanoPlot v1.46.2 [19]. Quality-filtered reads were mapped against the *B. fragilis* NCTC 9343 reference genome (RefSeq accession number GCF_000025985.1) using minimap2 v2.30 [20]. Per-base coverage was calculated with modsdepth v0.3.12 [21], yielding a mean coverage depth of 139.1× ± 48.75×. Raw sequencing reads were assembled using Hybracter v0.11.2 [22], and the sequencing assemblies were analyzed using Bactopia v3.2.0 [23]. Species identification was performed using the Genome Taxonomy Database Toolkit (GTDB-Tk) v2.4.0 [24], and the assembly quality was assessed using CheckM v1.2.3 [25]. Multi-locus sequence typing (MLST), which was based on seven core gene fragments (*groL*, *rpoB*, *recA*, *dnaJ*, *rprX*, *prfA* and *fusA*), was conducted using mlst v2.23.0 [26]. Antibiotic resistance genes were identified using AMRFinderPlus v4.0.19 [27]. For *cfiA*+ BF, the genomic location of *cfiA* was also determined by AMRFinderPlus, and the upstream region was searched for potential insertion sequences with reference to the ISfinder database (https://isfinder.biotoul.fr, last update: 21 November 2025, access date: 2 December 2025) [28]. The sequencing data of all isolates have been deposited in the National Center for Biological Information (NCBI) BioProject database under the accession number PRJNA1372458.

### 2.5. Phylogenetic Analysis

Phylogenetic analysis was performed to assess the genetic relationships among isolates. Core genomes were identified using a pangenome-based approach, and recombination events were masked prior to tree construction. A maximum-likelihood phylogenetic tree was then generated using IQ-Tree v2.2.2.7 [29] with 1000 ultrafast bootstrap replicates, and branches with bootstrap support ≥ 70% were considered well-supported. The resulting tree was visualized and annotated using Interactive Tree of Life (iTOL) v7.2.2.

### 2.6. Clinical Data Collection

Clinical information was retrospectively extracted from electronic medical records for all patients. The following information was included: demographics (age, gender, residential care home), medical history (recent surgery, use of immunosuppressive therapy, prior antibiotic exposure), comorbidities, clinical characteristics (presumed source of *B. fragilis* infections, temperature, co-infecting microorganisms), interventions (antibiotics, non-surgical drainage, surgery), ICU admission, duration of hospitalization, and mortality. The Charlson Comorbidity Index was calculated to stratify the comorbidity burden.

### 2.7. Definitions

Comorbidities were identified based on the information documented by the physicians in the medical records at the time of specimen collection. Chronic kidney disease was defined as having an estimated glomerular filtration rate (eGFR) less than 60 mL/min/1.73 m^2^ persisting for at least 3 months, or a history of renal transplant. Heart disease encompassed all cardiac conditions, including ischemic heart disease, congestive heart failure, arrhythmia, cardiomyopathy, and structural heart disease. Recent surgery, use of immunosuppressive therapy, and antibiotic exposure referred to any such events occurring within 4 weeks preceding specimen collection. Presumed source of infection was assigned for bacteremia cases according to the most plausible clinical site of origin based upon a review of medical records. Fever was defined as a body temperature exceeding 37.8 °C measured within 24 h of specimen collection. Co-infecting microorganisms referred to laboratory-confirmed microorganisms identified from the same specimen from which *B. fragilis* was isolated. Empirical antibiotic treatment was defined as antibiotic treatment administered during the time of specimen collection prior to the availability of culture results. It was considered appropriate if the corresponding isolate was later confirmed to be susceptible to at least one of the empirical antibiotics administered within 24 h of specimen collection by phenotypic testing. Early source control was defined as any surgical procedure or drainage performed within 4 days of specimen collection, or if an effective drainage system was already in place at the time of specimen collection. All-cause mortality was defined as death from any cause occurring within 7, 30, or 90 days after specimen collection.

### 2.8. Statistical Analysis

Data were analyzed using the IBM SPSS Statistics for Windows software, version 31.0 (IBM Corp., Armonk, NY, USA). Comparisons were made between (1) infections caused by *cfiA*+ BF and *cfiA*− BF, and (2) survivors and non-survivors based on 30-day outcomes. Categorical variables are expressed as frequency counts and percentages. Continuous variables are expressed as means +/− standard deviations (SDs) if normally distributed or as medians with interquartile ranges (IQRs) if non-normally distributed. Univariate analyses were conducted using the Chi-square test or Fisher’s exact test for categorical variables, and the Student’s *t*-test or the Mann–Whitney U-test for continuous variables, as appropriate. All variables with a *p*-value < 0.25 in univariate analyses were included in multivariate logistic regression to identify independent risk factors for *cfiA*+ BF infection and 30-day all-cause mortality. To avoid multicollinearity, variables of similar nature were not included simultaneously. The results are presented as odds ratios (ORs) with 95% confidence intervals (CIs). Two-tailed *p*-values < 0.05 were considered statistically significant. The *p*-values were reported as exploratory and were not adjusted for multiplicity.

## 3. Results

### 3.1. Isolate Information

A total of 166 non-duplicate *B. fragilis* isolates [40 (24.1%) *cfiA*-positive and 126 (75.9%) *cfiA*-negative] were retrieved. In total, 100 isolates (60.2%) were from United Christian Hospital (November 2023 to January 2025), 51 isolates (30.7%) were from Tseung Kwan O Hospital (January 2021 to January 2025), and 15 isolates (9.0%) were from Haven of Hope Hospital (January 2021 to January 2025). The isolates were most commonly obtained from blood (42.2%), followed by skin and soft tissue (32.5%) and intra-abdominal sources (16.3%) (Supplementary Table S1). The majority of *B. fragilis* infections were polymicrobial, with at least one co-infecting microorganism identified in 71.1% of cases. The most frequently isolated co-pathogens were *Escherichia coli* (33.1%), *Streptococcus anginosus* group (15.7%), *Staphylococcus aureus* (10.2%), and *Enterococcus* species (9.0%). *E. coli* co-infections were more common in *cfiA*+ BF infections (50.0% vs. 27.8%, *p* = 0.009), and *S. aureus* co-infections occurred more often in *cfiA*− BF infections (13.5% vs. 0%, *p* = 0.01) (Supplementary Table S2).

### 3.2. Genotypic Characteristics

**Figure 1:**
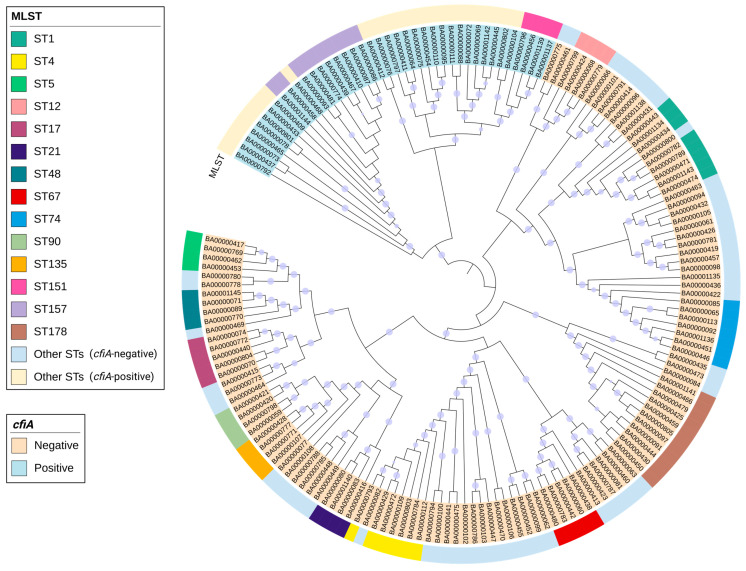
Maximum-likelihood phylogenetic tree of 166 *B. fragilis* isolates in Hong Kong based on core-genome alignment. The tree is midpoint-rooted, and branch lengths are not to scale. Branches with bootstrap support ≥70% are marked with purple dots. The strain numbers are prefixed with “BA”. Clades containing *cfiA*+ BF and *cfiA*− BF are shaded in blue and orange, respectively. The outer circle represents multi-locus sequencing (MLST) sequence types. Sequence types with ≥4 isolates are assigned specific color annotations, while all remaining sequence types are grouped as “Other STs (*cfiA*-negative)” and “Other STs (*cfiA*-positive)”.

The 166 *B. fragilis* isolates belonged to 81 different sequence types (STs). Phylogenetic analysis showed that the strains were clustered into two major divisions, I and II, corresponding to *cfiA* status. The *cepA* gene was found in all except one isolate (99.2%) belonging to division I. Based on MLST, isolates sharing the same STs consistently belonged to the same division. However, within each division, isolates exhibited considerable genetic diversity. In division I (*cfiA*− BF), the most prevalent STs were ST178 (*n* = 11), ST1 (*n* = 8), ST4 (*n* = 7), ST74 (*n* = 7), ST17 (*n* = 5), and ST67 (*n* = 5). In division II (*cfiA*+ BF), the predominant STs were ST157 (*n* = 10), ST151 (*n* = 4), and ST52 (*n* = 3) (Figure 1). A total of 18 isolates (10.8%) carried the *bft* gene, all of which belonged to division I (*cfiA*− BF), with *bft-1* being the predominant subtype. No specific association was observed between the ST and infection sites.

For the 40 division II (*cfiA*+ BF) isolates, upstream insertion sequences were identified in 19 of them (47.5%), all located within 100 bp of the *cfiA* start codon. The most prevalent insertion sequence type was IS*1187* (*n* = 11), which was present in 70.0% of ST157 isolates and also detected in four other sequence types. The second and third most common insertion sequence types were IS*613* (*n* = 4) and IS*616* (*n* = 2), respectively (Supplementary Table S3).

The distribution of 29 antibiotic resistance genes conferring resistance to 7 classes of antibiotics among the *B. fragilis* isolates is summarized in Supplementary Table S4. The most prevalent resistance gene was *tetQ* (90.4%), followed by *cepA* (75.3%) and *ermF* (55.4%). On the other hand, the *nimJ* (1.2%), *nimA* (0.6%), and *nimE* (0.6%) genes for nitroimidazole resistance were rare. A small proportion of isolates, in both divisions I and II, harbored emerging beta-lactamase genes *bla*_OXA-347_ (6.0%), *bla*_MUN-1_ (3.0%), and *bla*_MUN-5_ (0.6%). When comparing between *cfiA*+ BF and *cfiA*− BF isolates, *lnu(AN2)* (18.3% vs. 5.0%; *p* = 0.04) and *mef(En2)* (18.3% vs. 5.0%; *p* = 0.04) were detected more often in *cfiA*− BF isolates. In contrast, *bla*_OXA-347_ (12.5% vs. 4.0%; *p* = 0.06) and *erm(F)* (67.5% vs. 51.6%; *p* = 0.08) tended to occur more frequently in *cfiA*+ BF isolates.

### 3.3. Phenotypic Antibiotic Resistance and Association with cfiA Status

According to the EUCAST clinical breakpoint guideline, more than half of the *B. fragilis* isolates exhibited resistance to amoxicillin–clavulanic acid (52.4%), ampicillin–sulbactam (51.8%), and clindamycin (51.2%). The rates of resistance to ertapenem (28.9%) and meropenem (27.7%) were higher than those to imipenem (16.3%). The lowest resistance rate was observed for metronidazole (13.9%).

The detection of *cfiA* using the MALDI-TOF MS Bruker Biotyper MBT subtyping IVD module demonstrated 100% concordance with the Oxford Nanopore sequencing results. A positive *cfiA* result was strongly associated with phenotypic carbapenem resistance, showing the highest correlation with meropenem (Φ = 0.82, *p* < 0.001) and the lowest with imipenem (Φ = 0.63, *p* < 0.001). A moderate association was also detected between *cfiA* positivity and phenotypic resistance to beta-lactam and beta-lactamase inhibitor combinations, with the strongest association observed for piperacillin–tazobactam (Φ = 0.43, *p* < 0.001). Phenotypically, *cfiA*+ BF isolates, especially those belonging to ST157, exhibited marked resistance to most antibiotics, except metronidazole. The level of carbapenem resistance differed with respect to the presence of upstream insertion sequences. All *cfiA*+ BF isolates with upstream insertion sequences exhibited phenotypic resistance to all three carbapenems, whereas 90.5% of isolates without such sequences remained phenotypically susceptible to at least one carbapenem (Supplementary Table S3). A similarly high level of resistance was also observed for isolates harboring *bla*_OXA-347_ and *bla*_MUN-1_. Conversely, the majority of *cfiA*− BF isolates were phenotypically susceptible to piperacillin–tazobactam (87.3%), carbapenems (88.9% to ertapenem; 96.8% to imipenem; 92.9% to meropenem), and metronidazole (84.9%) (Table 1). Three isolates (1.8%) displayed concomitant resistance to all three carbapenems and metronidazole. Furthermore, two of these isolates (1.2%) were resistant to all eight antibiotics tested in the study. Both were division II isolates, one belonging to ST157 and the other to ST202.

### 3.4. Clinical Characteristics of Patients with cfiA+ BF Infection

The median age of the patients was 73.0 (IQR 60.3–85.0), with the majority (65.7%) aged over 65. Slightly more female (53.0%) than male patients (47.0%) were included. There were no significant differences in terms of age and sex distribution with respect to *cfiA* status. Approximately half (48.8%) of the patients had a Charlson Comorbidity Index more than or equal to five. Common comorbidities included diabetes mellitus (35.5%), chronic kidney disease (24.1%) and active malignancy (21.1%). A greater proportion of patients with *cfiA*+ BF infections had a history of recent surgery (10.0% vs. 3.2%; *p* = 0.10) compared to those with *cfiA*− BF infections, although the difference was not statistically significant. No significant differences were observed between the *cfiA*+ BF and *cfiA*− BF groups regarding the presumed sources of infection or the presence of fever (Supplementary Table S5). Univariate analysis identified diabetes mellitus as a potential risk factor (*p* = 0.03) for *cfiA*+ BF infection. However, multivariate logistic regression did not identify any independent risk factors (Supplementary Table S6).

Most of the patients (84.9%) had received empirical antibiotics with expected activity against *B. fragilis*, primarily amoxicillin–clavulanic acid (56.0%) and piperacillin–tazobactam (17.5%). Appropriate empirical antibiotic use was less common in those with *cfiA*+ BF infections (43.6% vs. 55.6%; *p* = 0.19). Regarding targeted therapy, the most widely prescribed antibiotics were amoxicillin–clavulanic acid (36.1%), metronidazole (31.9%), and piperacillin–tazobactam (22.3%). Meropenem was used more often as targeted therapy (15.1%), particularly in those with *cfiA*+ BF infections (25.0%). Similarly, appropriate use of targeted antibiotics was less frequent in *cfiA*+ BF infections (62.5% vs. 75.4%; *p* = 0.11). The 30-day all-cause mortality following *B. fragilis* infection was 13.3%. Patients with *cfiA*+ BF infections appeared to have a higher likelihood of ICU admission (12.5% vs. 6.3%; *p* = 0.31) and prolonged hospital stay (40.5% vs. 27.5%; *p* = 0.13), and increased 7-day (7.5% vs. 3.2%; *p* = 0.36), 30-day (17.5% vs. 11.9%; *p* = 0.36), and 90-day (25.0% vs. 23.8%; *p* = 0.88) all-cause mortality, although none of these differences reached statistical significance (Table 2).

### 3.5. Predictors for 30-Day All-Cause Mortality Following B. fragilis Infection

Patients with *B. fragilis* infections were classified into survivor and non-survivor groups by their 30-day outcomes. Univariate analysis showed that the risk factors for 30-day all-cause mortality included a high Charlson Comorbidity Index (*p* < 0.001), active malignancy (*p* < 0.001), and the absence of early source control (*p* < 0.001). The mortality rate appeared to be similar regardless of the appropriateness of empirical or targeted antibiotics (Table 3). Multivariate logistic regression revealed that a high Charlson Comorbidity Index [OR = 1.30; 95% confidence interval (CI): 1.04–1.63, *p* = 0.02] and the absence of early source control [OR = 4.84; 95% confidence interval (CI): 1.18–19.75, *p* = 0.03] were independent risk factors for 30-day all-cause mortality (Table 4).

## 4. Discussion

*B. fragilis* is the leading cause of severe anaerobic infections, but the emergence of antibiotic resistance has complicated the selection of effective antibiotic therapy. The MALDI-TOF MS Bruker Biotyper MBT subtyping IVD module has demonstrated potential as a rapid and reliable tool for the detection of *cfiA*, the predominant carbapenemase gene in *B. fragilis*. In this study, we investigated the clinical significance of *cfiA*+ BF detection and evaluated the patient characteristics and outcomes associated with these infections.

### 4.1. MALDI-TOF MS as a Rapid and Useful Diagnostic Tool for cfiA Detection

Consistent with previous studies [12,13,14,15], the detection of *cfiA* using the MALDI-TOF MS Bruker Biotyper MBT subtyping IVD module demonstrated excellent concordance (100%) with gene sequencing results, confirming its reliability as a rapid diagnostic tool. The *cfiA* positivity correlated strongly with phenotypic carbapenem resistance and moderately with beta-lactam and beta-lactamase inhibitor combination resistance. Among individual carbapenems, the association was strongest for meropenem and weakest for imipenem. For example, while only 7.5% of *cfiA*+ BF isolates were phenotypically susceptible to meropenem, up to 42.5% of them were susceptible to imipenem. Similar patterns of differential carbapenem resistance have been reported in other studies [30], in which the minimal inhibitory concentrations (MICs) of *cfiA*+ BF were generally lower for imipenem than for meropenem. This difference may be related to the more efficient hydrolysis of meropenem by beta-lactamases [15,31]. Accordingly, meropenem may serve as a more sensitive phenotypic marker of *cfiA*-mediated resistance than imipenem [32].

Approximately half (47.5%) of the *cfiA*+ BF isolates in our cohort possessed upstream insertion sequences, and all of them demonstrated phenotypic resistance to all three carbapenems tested. These insertion sequence types are similar to those previously described in Hong Kong [33]. They are thought to act as strong promoters for *cfiA* expression and thereby confer high-level carbapenem resistance. In contrast, 90.5% of *cfiA*+ BF isolates without upstream insertion sequences displayed only partial carbapenem resistance, probably related to the low level of constitutive *cfiA* expression. Their resistance may also be mediated by other genetic determinants such as porin loss and the upregulation of efflux pumps [10,30,34,35,36,37]. Although such isolates may appear phenotypically susceptible, carbapenem should be used with caution in these cases, because insertion sequences can be acquired over time, and exposure to carbapenem may select for hetero-resistant mutants [38]. In addition, two isolates without detectable upstream insertion sequences also demonstrated high-level carbapenem resistance, which may be due to novel insertion sequences, other resistance genes, or the presence of efflux pumps [39]. At present, clinical data regarding the implications of the above microbiological findings are limited, and further studies incorporating genomic and functional analysis are warranted to elucidate the resistance mechanism and their clinical relevance. Nonetheless, it seems reasonable to consider alternative classes of antibiotics in all cases with *cfiA*+ BF infection. Metronidazole, for instance, may be considered due to its relatively low resistance. On the other hand, carbapenems remain as a suitable empirical therapy for *cfiA*− BF isolates, which are typically susceptible to carbapenems, as well as to piperacillin–tazobactam and metronidazole. When selecting empirical treatment for *B. fragilis* infections, clinicians should also consider the source of infection, disease severity, and likely co-pathogens. For example, piperacillin–tazobactam and meropenem may better penetrate into intra-abdominal abscesses than imipenem and ertapenem [40].

### 4.2. High Antibiotic Resistance and Risk of Multidrug Resistance in B. fragilis

Our study revealed an overall high prevalence of antibiotic resistance in *B. fragilis*. Among the 166 isolates, 40 (24.1%) were *cfiA*-positive. This prevalence is comparable to findings from Hong Kong [16] and mainland China [14], but higher than that reported in Europe [41]. *B. fragilis* ST157, which is usually found in Asia and characterized by marked carbapenem resistance [42], accounted for 25.0% of *cfiA*+ BF in our cohort. Nearly two-thirds (63.6%) of the isolates expressed high-level carbapenem resistance in the presence of upstream IS*1187.* Although other studies have also identified IS*1187* in *cfiA*+ BF, direct comparison of the prevalence is difficult because of heterogeneity in sample types and study designs [33,43,44]. Phenotypically, more than half of the *B. fragilis* isolates exhibited resistance to amoxicillin–clavulanic acid, ampicillin–sulbactam, and clindamycin, with rates notably higher than those reported in previous studies [4,45,46,47].

The emergence of *B. fragilis* with multi-drug resistance in our cohort may pose additional challenges in clinical management. Three isolates showed concomitant resistance to metronidazole and all three carbapenems, and two of them even exhibited resistance to all eight antibiotics tested. This extensive pattern of resistance has been rare and mainly described in case reports. Treatment options for these isolates are limited, and the optimal antibiotic therapy remains uncertain [48,49].

The increased resistance observed in our cohort may be partly attributable to differences in testing methodologies and interpretative breakpoints. While previous studies typically assessed *B. fragilis* phenotypic susceptibility using the agar dilution or gradient diffusion method based on the Clinical and Laboratory Standard Institute (CLSI) or earlier EUCAST guidelines for anaerobes, we employed the disk diffusion method in accordance with the EUCAST 2025 guidelines because it provides species-specific breakpoints for *B. fragilis* and is less labor-intensive to perform. Traditionally, disk diffusion was not recommended for anaerobic sensitivity testing because it showed substantial variability in zone diameters and poor agreement with the reference methodology [50,51]. However, the new EUCAST disk diffusion method for anaerobic sensitivity has demonstrated an excellent overall categorical agreement with the CLSI agar dilution method, although its breakpoints appeared to be more stringent [52,53]. While the change in methodology may have contributed to the higher resistance rates, it is noteworthy that the CLSI breakpoints for anaerobes have remained largely unchanged for more than a decade. In contrast, the EUCAST disk diffusion method was recently evaluated, with breakpoints specific to *B. fragilis* introduced [54]. The higher resistance rates observed in our cohort may therefore reflect a genuine rise and should not be easily dismissed. Although our sample size was modest and derived from a single region, clinicians should be cautious of this apparent increasing trend of resistance and revise their empirical antibiotic regimes based on the latest local epidemiology.

### 4.3. Emerging Beta-Lactamase Genes Other than cfiA

While *cfiA* remains a key antibiotic resistance gene in *B. fragilis*, our study also identified other emerging beta-lactamase genes, including *bla*_OXA-347_ (6.0%), *bla*_MUN-1_ (3.0%) and *bla*_MUN-5_ (0.6%), which were detected in both phylogenetic divisions. The *bla*_OXA-347_ gene, encoding a type D beta-lactamase, was first reported in *B. fragilis* in 2015 and has also been found in Enterobacteriaceae and Flavobacteriaceae. It has been widely detected in fecal samples from both humans and animals, as well as in wastewater. Its association with mobile transposons has raised concern regarding inter-species horizontal gene transfer [55,56,57,58]. Meanwhile, the *bla*_MUN_ gene encodes a type A beta-lactamase with extended spectrum beta-lactamase (ESBL)-like activity. Although *bla*_MUN-1_ was initially described in *Bacteroides* species, it has also been identified in *Sutterella wadsworthensis*, a member of the phylum Pseudomonadota, raising suspicion of possible interphylum transfer [59]. In our cohort, isolates harboring either *bla*_OXA-347_ or *bla*_MUN-1_ exhibited a resistance pattern similar to *cfiA*+ BF, showing increased resistance to most antibiotics except metronidazole. The emergence of these genes indicates ongoing diversification of resistance mechanisms in *B. fragilis* and reinforces the need for phenotypic susceptibility confirmation. Moreover, their potential for horizontal transfer is concerning due to the risk of resistance dissemination to other species and the environment. Continuous surveillance is therefore crucial to identify ongoing resistance threats.

### 4.4. cfiA+ BF Infections Associated with Higher Risk of Suboptimal Treatment and Possible Adverse Outcomes

In addition to microbiological and genotypic analysis of *cfiA*+ BF, our study also analyzed the clinical characteristics and outcomes of patients with these infections. In our cohort, the majority of patients with *B. fragilis* infections were aged 65 years or older and had a high Charlson Comorbidity Index, reflecting a population with substantial baseline frailty and comorbidities. Diabetes mellitus appeared to be associated with *cfiA*+ BF infection in univariate analysis, but this relationship was not maintained in multivariate logistic regression. The absence of independent clinical predictors suggested that risk factors for *cfiA*+ BF infection may be multifactorial or environmentally influenced, and MALDI-TOF MS is an invaluable tool for the rapid and accurate detection of these strains, especially in high-prevalence regions.

The emergence of *cfiA*+ BF strains poses challenges in antibiotic selection. Even though most patients received empirical and targeted antibiotics with expected activity against *B. fragilis*, a substantial proportion of these antibiotics were considered inappropriate based on phenotypic testing, especially those with *cfiA*+ BF infections. This finding suggests that the current practice of choosing an empirical antibiotic regime based on expected susceptibility may not be able to address the evolving resistance patterns. Additional genotypic and phenotypic susceptibility tests should be performed, especially for patients with severe infections.

We also evaluated the outcomes of patients with *cfiA*+ BF infections. Despite not reaching statistical significance, these infections were associated with a trend toward higher ICU admission rates, prolonged hospitalization, and increased all-cause mortality at 7, 30, and 90 days. Although our sample size was modest, these findings suggest that *cfiA*+ BF infections may be associated with adverse clinical outcomes, and that early detection may assist in guiding appropriate patient management.

### 4.5. Early Source Control May Improve the Management of B. fragilis Infections

Another notable observation is that the 30-day all-cause mortality following *B. fragilis* infection was higher among patients with substantial baseline comorbidities and in those without early source control. The importance of source control has been well-established in the management of infections that frequently involve anaerobes, such as intra-abdominal infections [60,61,62]. This is critical because anaerobic infections often result in necrotic tissue or abscess formation, where antibiotic penetration is limited. The low pH and the inactivating enzymes inside an abscess may further impair antibiotic activity [63]. Early source control reduces the bacterial load and improves the effectiveness of antibiotic therapy.

The appropriateness of antibiotic therapy was not identified as an independent predictor of 30-day all-cause mortality in our study, consistent with several other studies. Blairon et al. reported that the severity of underlying disease and the immunosuppression status were more strongly associated with mortality than the effectiveness of antimicrobial therapy [64], whereas Kovács et al. identified advanced age as the major predictor [65]. However, other studies [66,67,68], including a prospective multicenter observational study [5], have demonstrated a significant association between appropriate antimicrobial therapy and improved outcomes. Despite these conflicting results, the clinical importance of appropriate antibiotic therapy in optimizing outcomes of *B. fragilis* infection should not be overlooked. The discrepancies among studies may reflect variations in patient populations, sample sizes, study designs, and the adequacy of source control. In our cohort, for instance, the impact of antibiotic appropriateness may have been attenuated by the substantial comorbidity burden observed among patients.

### 4.6. Strengths and Limitations

The strength of this study lies in its integrated clinical, microbiological, and genotypic analysis for *cfiA*+ BF infections over a four-year period within an urban region. The number of infections was considerable, and data were identified for the vast majority of clinical and microbiological variables. However, several important limitations should be acknowledged. First, as a retrospective observational study, the analysis relied on existing medical records that may be incomplete or subject to information bias, and causal inferences may not be firmly established. In particular, some variables, such as the timing and adequacy of source control, may not have been consistently recorded. This could have led to misclassification and introduced bias into the observed associations. Nevertheless, the retrospective design enabled standardized data extraction, and our resulting dataset was sufficiently comprehensive to support statistical analysis. Second, the sample size was modest and limited to three hospitals, which may have reduced the statistical power and limited the generalizability of the findings to other populations. There was also a potential risk of selection bias because strain-saving practices differed across individual laboratories, and only culture-confirmed cases were included. Still, this study provides a useful summary of regional data by analyzing isolates from multiple hospitals and may offer insights relevant to regions with a similarly high prevalence of *cfiA*+ BF. Third, the appropriateness of antibiotic therapy was based on phenotypic in vitro susceptibility without consideration of other co-pathogens and pharmacokinetic factors such as tissue penetration. However, even under these favorable assumptions, a substantial proportion of patients still received inappropriate therapy based on phenotypic testing, suggesting that the problem may be even more pronounced in real-world practice. Fourth, a less stringent cutoff for variable selection has been used to allow for a more exploratory analysis because the number of previous studies was limited. Although this may increase the risk of overfitting, we identified potential predictors for 30-day all-cause mortality with statistical significance, which may warrant further analysis. Finally, a functional assay assessing resistance gene expression was not performed; hence, the exact relationship between the *cfiA* gene, upstream insertion sequences, and carbapenemase activity could not be clearly defined. Nonetheless, most of the insertion sequences detected in our cohort have been shown to possess promoter activity in *cfA*+ BF. Moreover, the strong correlation between *cfiA* and phenotypic carbapenem resistance, as well as the differential carbapenem resistance pattern observed among *cfiA*+ BF, is consistent with previous studies. In the future, large-cohort prospective studies assessing clinical outcomes when therapy is adjusted based on MALDI-TOF MS *cfiA* results, together with functional studies of *cfiA* expression and carbapenemase activity, would be valuable.

## 5. Conclusions

This study highlights the high prevalence of *cfiA*+ BF in Hong Kong and underscores the clinical significance of rapid *cfiA* detection using MALDI-TOF MS. A positive *cfiA* result was strongly correlated with phenotypic carbapenem resistance and moderately correlated with resistance to beta-lactam and beta-lactamase inhibitor combinations. A high rate of antibiotic resistance in *B. fragilis* was observed, accompanied by the emergence of new beta-lactamase genes. The detection of multidrug-resistant strains is particularly concerning, as it may further limit available treatment options. Continuous genotypic and phenotypic surveillance is recommended to monitor resistance trends. Incorporation of *cfiA* detection using MALDI-TOF MS into the diagnostic workflow for *B. fragilis* isolates may be beneficial in high-prevalence regions. When *cfiA*+ BF infection is confirmed, carbapenems should generally be avoided and alternative agents such as metronidazole may be considered, in conjunction with effective source control. Given the retrospective, single-region design of our study, these conclusions should be interpreted with caution, and validation in further large-scale prospective studies and meta-analyses is warranted.

## Figures and Tables

**Table 1:** Association between phenotypic antibiotic resistance and *cfiA* status of *B. fragilis* isolates.

Antibiotic	All Isolates (*n* = 166)	*cfiA*+ BF (*n* = 40)	*cfiA*− BF (*n* = 126)	Phi Coefficient (Φ)	*p*-Value ^1^
	Number (Percent)				
Amoxicillin–clavulanic acid	87 (52.4)	32 (80.0)	55 (43.7)	0.31	<0.001
Ampicillin–sulbactam	86 (51.8)	35 (87.5)	51 (40.5)	0.40	<0.001
Piperacillin–tazobactam	38 (22.9)	22 (55.0)	16 (12.7)	0.43	<0.001
Ertapenem	48 (28.9)	34 (85.0)	14 (11.1)	0.70	<0.001
Imipenem	27 (16.3)	23 (57.5)	4 (3.2)	0.63	<0.001
Meropenem	46 (27.7)	37 (92.5)	9 (7.1)	0.82	<0.001
Metronidazole	23 (13.9)	4 (10.0)	19 (15.1)	0.06	0.42
Clindamycin	85 (51.2)	25 (62.5)	60 (47.6)	0.13	0.10

Abbreviations: *cfiA*+ BF, *cfiA*-positive *Bacteroides fragilis*; *cfiA*− BF, *cfiA*-negative *Bacteroides fragilis*. ^1^ *p*-values were calculated by comparing the *cfiA*+ BF with the *cfiA*− BF groups.

**Table 2:** Treatment and clinical outcomes of patients with *B. fragilis* infections.

	Variable	All Patients (*n* = 166)	*cfiA*+ BF (*n* = 40)	*cfiA*− BF (*n* = 126)	*p*-Value ^1^
		Number (Percent)			
Empirical antibiotics	Amoxicillin–clavulanic acid	93 (56.0)	19 (47.5)	74 (58.7)	0.21
	Piperacillin–tazobactam	29 (17.5)	6 (15.0)	23 (18.3)	0.64
	Cefoperazone–sulbactam	1 (0.6)	1 (2.5)	0	0.24
	Meropenem	8 (4.8)	3 (7.5)	5 (4.0)	0.40
	Ertapenem	4 (2.4)	1 (2.5)	3 (2.4)	1.00
	Metronidazole	10 (6.0)	5 (12.5)	5 (4.0)	0.06
	Clindamycin	1 (0.6)	1 (2.5)	0	0.24
	Absence of empirical antibiotics with expected activity against *B. fragilis*	25 (15.1)	6 (15.0)	19 (15.1)	0.99
	Appropriate empirical antibiotics ^2^	87/165 (52.7)	17/39 (43.6)	70 (55.6)	0.19
Targeted antibiotics	Amoxicillin–clavulanic acid	60 (36.1)	12 (30.0)	48 (38.1)	0.35
	Piperacillin–tazobactam	37 (22.3)	9 (22.5)	28 (22.2)	0.97
	Cefoperazone–sulbactam	1 (0.6)	0	1 (0.79)	1.00
	Meropenem	25 (15.1)	10 (25.0)	15 (11.9)	0.04
	Ertapenem	9 (5.4)	1 (2.5)	8 (6.3)	0.69
	Metronidazole	53 (31.9)	11 (27.5)	42 (33.3)	0.49
	Clindamycin	4 (2.4)	1 (2.5)	3 (2.4)	1.00
	Absence of targeted antibiotics with expected activity against *B. fragilis*	14 (8.4)	5 (12.5)	9 (7.1)	0.33
	Appropriate targeted antibiotics	120 (72.3)	25 (62.5)	95 (75.4)	0.11
Interventions	Early source control	84 (50.6)	21 (52.5)	63 (50.0)	0.78
	ICU admission	13 (7.8)	5 (12.5)	8 (6.3)	0.31
Outcome	Duration of hospital stay ≥ 30 days ^3^	48/157 (30.6)	15/37 (40.5)	33/120 (27.5)	0.13
All-cause mortality	7 days	7 (4.2)	3 (7.5)	4 (3.2)	0.36
	30 days	22 (13.3)	7 (17.5)	15 (11.9)	0.36
	90 days	40 (24.1)	10 (25.0)	30 (23.8)	0.88

Abbreviations: *cfiA*+ BF, *cfiA*-positive *Bacteroides fragilis*; *cfiA*− BF, *cfiA*-negative *Bacteroides fragilis*. ^1^ *p*-values were calculated by comparing the *cfiA*+ BF with the *cfiA*− BF groups. ^2^ One patient (*cfiA*+ BF) treated with cefoperazone–sulbactam only was excluded because the appropriateness of therapy could not be determined due to the absence of an EUCAST clinical breakpoint. ^3^ Nine outpatient patients (three *cfiA*+ BF and six *cfiA*− BF) were excluded.

**Table 3:** Univariate analysis of predictors for 30-day all-cause mortality following *B. fragilis* infections.

	Variable	All Patients (*n* = 166)	Deceased (*n* = 22)	Survivors (*n* = 144)	*p*-Value ^1^
		Number (Percent)			
Demographics	Median age (IQR)	73.0 (60.3–85.0)	77.0 (63.5–85.0)	72.5 (58.8–85.3)	0.23
	Age ≥ 65	109 (65.7)	16 (72.7)	93 (64.6)	0.45
	Male gender	78 (47.0)	11 (50.0)	67 (46.5)	0.76
	Residential care home	24 (14.5)	4 (18.2)	20 (13.9)	0.53
Charlson Comorbidity Index	Median score (IQR)	4.0 (2.0–6.8)	7.5 (6.0–8.0)	4.0 (2.0–6.0)	<0.001
	0	25 (15.1)	0	25 (17.4)	
	1–2	18 (10.8)	1 (4.5)	17 (11.8)	
	3–4	42 (25.3)	1 (4.5)	41 (28.5)	
	≥ 5	81 (48.8)	20 (90.9)	61 (42.4)	
Comorbidities	Diabetes mellitus	59 (35.5)	11 (50.0)	48 (33.3)	0.13
	Active malignancy	35 (21.1)	13 (59.1)	22 (15.3)	<0.001
	Chronic kidney disease	40 (24.1)	7 (31.8)	33 (22.9)	0.36
	Liver cirrhosis	1 (0.6)	0	1 (0.7)	1.00
	Heart disease	32 (19.3)	5 (22.7)	27 (18.8)	0.77
Medical history	Recent surgery	8 (4.8)	3 (13.6)	5 (3.5)	0.07
	Recent immunosuppressant use	10 (6.0)	3 (13.6)	7 (4.9)	0.13
Antibiotic exposure	Recent antibiotic use	51 (30.7)	10 (45.5)	41 (28.5)	0.11
	Carbapenem	7 (4.2)	1 (4.5)	6 (4.2)	1.00
	Beta-lactam/beta-lactamase inhibitor combination	33 (19.9)	5 (22.7)	28 (19.4)	0.77
Antibiotic treatment	Appropriate empirical antibiotics ^2^	87/165 (52.7)	12 (54.5)	75/143 (52.4)	0.85
	Appropriate targeted antibiotics	120 (72.3)	16 (72.7)	104 (72.2)	0.96
Presumed source of infection	Abdomen	64 (38.6)	12 (54.5)	52 (36.1)	0.10
	Skin and soft tissue	56 (33.7)	2 (9.1)	54 (37.5)	0.009
	Genital tract	16 (9.6)	1 (4.5)	15 (10.4)	0.70
	Septicemia with uncertain source	23 (13.9)	6 (27.3)	17 (11.8)	0.09
	Others	7 (4.2)	1 (4.5)	6 (4.2)	1.00
Clinical characteristics	Fever (> 37.8 °C) ^3^	68/158 (43.0)	12 (54.5)	56/136 (41.2)	0.24
	*cfiA*+ BF	40 (24.1)	7 (31.8)	33 (22.9)	0.36
Interventions	Absence of early source control	82 (49.4)	19 (86.4)	63 (43.8)	<0.001
	ICU admission	13 (7.8)	2 (9.1)	11 (7.6)	0.68

^1^ *p*-values were calculated by comparing the *cfiA*+ BF with the *cfiA*− BF groups. ^2^ One patient (survivor) treated with cefoperazone–sulbactam only was excluded because the appropriateness of therapy could not be determined due to the absence of an EUCAST clinical breakpoint. ^3^ Eight patients (all survivors) without temperature records were excluded.

**Table 4:** Multivariate logistic regression for predictors of 30-day all-cause mortality following *B. fragilis* infections.

Variable	Odds Ratio (OR)	95% Confidence Interval (95% CI)	*p*-Value
Age	0.99	0.96–1.03	0.70
Charlson Comorbidity Index	1.30	1.04–1.63	0.02
Recent surgery	0.40	0.06–2.87	0.36
Recent immunosuppressant use	0.71	0.11–4.76	0.72
Recent antibiotic use	1.28	0.39–4.22	0.68
Presumed intra-abdominal infections	2.04	0.38–12.68	0.45
Presumed skin and soft tissue infections	0.57	0.06–5.20	0.62
Septicemia with uncertain source	1.71	0.23–12.55	0.60
Fever	0.91	0.30–2.74	0.86
Absence of early source control	4.84	1.18–19.75	0.03

## Data Availability

The original contributions presented in this study are included in the article/Supplementary Materials. Further inquiries can be directed to the corresponding author.

## Supplementary Materials

The following supporting information can be downloaded at: https://www.mdpi.com/article/10.3390/microorganisms14010168/s1. Table S1: Types of specimens from which *B. fragilis* was isolated; Table S2: Ten most common co-infecting microorganisms in *B. fragilis* infections; Table S3: Summary of upstream insertion sequences of *cfiA*-positive *B. fragilis*; Table S4: Distribution of antibiotic resistance genes; Table S5: Baseline demographics and clinical characteristics of patients with *B. fragilis* infections; Table S6: Multivariate logistic regression for risk factors of *cfiA*-positive *B. fragilis* infections; Table S7: Treatment and clinical outcomes of patients with *B. fragilis* infections.

## Abbreviations

The following abbreviations are used in this manuscript:

*cfiA*+ BF	*cfiA*-positive *Bacteroides fragilis*
*cfiA*− BF	*cfiA*-negative *Bacteroides fragilis*
MLST	Multi-locus sequence typing
ST	Sequence type
IQR	Interquartile range
OR	Odds ratio
CI	Confidence interval
