# Supplementary material for: Clinical Significance of cfiA Positivity Detected by Matrix-Assisted Laser Desorption/Ionization Time-of-Flight Mass Spectrometry in Bacteroides fragilis Infections
Source: Microorganisms. 2026 Jan 12;14(1):168. doi: 10.3390/microorganisms14010168 (PMC12844121; doi:10.3390/microorganisms14010168)
Supplement: Supplementary file 1 [file microorganisms-14-00168-s001.zip › Supplementary Table S1 S2 S4 S5 S6 S7 cfiA Bacteroides fragilis V2.4.pdf]

microorganisms

MDPI

Supplementary Table S1. Types of specimens from which *B. fragilis* was isolated.

Specimen Type	All Isolates (n = 166)	*cfiA*+ BF (n = 40)	*cfiA*− BF (n = 126)	p-Value [1]
	Number (Percent)			
Blood	70 (42.2)	17 (42.5)	53 (42.1)	0.96
Skin and soft tissue	54 (32.5)	13 (32.5)	41 (32.5)	1.00
Intra-abdominal	27 (16.3)	4 (10.0)	23 (18.3)	0.22
Genital tract	12 (7.2)	5 (12.5)	7 (5.6)	0.16
Others	3 (1.8)	1 (2.5)	2 (1.6)	0.57

Abbreviations: *cfiA*+ BF, *cfiA*-positive *Bacteroides fragilis*; *cfiA*− BF, *cfiA*-negative *Bacteroides fragilis*. [1] p-values were calculated by comparing the *cfiA*+ BF with the *cfiA*− BF groups.

Supplementary Table S2. Ten most common co-infecting microorganisms in *B. fragilis* infections.

Co-Infecting Microorganism	All Isolates (*n* = 166)	*cfiA*+ BF (*n* = 40)	*cfiA*− BF (*n* = 126)	*p*-Value [1]
	Number (Percent)			
Escherichia coli	55 (33.1)	20 (50.0)	35 (27.8)	0.009
Streptococcus anginosus group	26 (15.7)	10 (25.0)	16 (12.7)	0.06
Staphylococcus aureus	17 (10.2)	0	17 (13.5)	0.01
Enterococcus species	15 (9.0)	5 (12.5)	10 (7.9)	0.36
Klebsiella pneumoniae complex	13 (7.8)	2 (5.0)	11 (8.7)	0.74
*Pseudomonas aeruginos*a	10 (6.0)	3 (7.5)	7 (5.6)	0.71
Proteus species	8 (4.8)	2 (5.0)	6 (4.8)	1.00
Fusobacterium species	8 (4.8)	3 (7.5)	5 (4.0)	0.40
Bacteroides species	6 (3.6)	0	6 (4.8)	0.34
Peptostreptococcus species	5 (3.0)	1 (2.5)	4 (3.2)	1.00

Abbreviations: *cfiA*+ BF, *cfiA*-positive *Bacteroides fragilis*; *cfiA*− BF, *cfiA*-negative *Bacteroides fragilis*. [1] *p*-values were calculated by comparing the *cfiA*+ BF with the *cfiA*− BF groups.

Supplementary Table S4. Distribution of antibiotic resistance genes.

Antibiotic Class	Resistance Gene	All Isolates (*n* = 166)	*cfiA*+ BF (*n* = 40)	*cfiA*− BF (*n* = 126)	*p*-Value [1]
			Number (Percent)		
Beta-lactam	*cepA*	125 (75.3)	0	125 (99.2)	<0.001
	cfxA	59 (35.5)	13 (32.5)	46 (36.5)	0.64
	cfiA	40 (24.1)	40 (100.0)	0	<0.001
	$bla_{OXA-347}$	10 (6.0)	5 (12.5)	5 (4.0)	0.06
	bla_{MUN-1}	5 (3.0)	2 (5.0)	3 (2.4)	0.60
	bla_{MUN-5}	1 (0.6)	0	1 (0.8)	1.00
Aminoglycoside	*aadE*	18 (10.8)	4 (10.0)	14 (11.1)	1.00
	aad9	17 (10.2)	4 (10.0)	13 (10.3)	1.00
	aph(3′)-IIIa	14 (8.4)	3 (7.5)	11 (8.7)	1.00
	ant(9)	14 (8.4)	2 (5.0)	12 (9.5)	0.52
	ant(6)-Ia	12 (7.2)	2 (5.0)	10 (7.9)	0.73
	aadS	10 (6.0)	3 (7.5)	7 (5.6)	0.71
	aph(3″)-Ib	5 (3.0)	1 (2.5)	4 (3.2)	1.00
	aph(6)-Id	2 (1.2)	0	2 (1.6)	1.00
	aac(6′)-Ie/aph(2″)-Ia	2 (1.2)	0	2 (1.6)	1.00
Lincosamide/macrol-ide/streptogramin	*erm(F)*	92 (55.4)	27 (67.5)	65 (51.6)	0.08
	erm(G)	2 (1.2)	0	2 (1.6)	1.00
Lincosamide	*lnu(AN2)*	25 (15.1)	2 (5.0)	23 (18.3)	0.04
Macrolide	*mef(En2)*	25 (15.1)	2 (5.0)	23 (18.3)	0.04
	mef(A)	16 (9.6)	3 (7.5)	13 (10.3)	0.76
	estT	7 (4.2)	0	7 (5.6)	0.20
	ere(D)	5 (3.0)	3 (7.5)	2 (1.6)	0.09
Tetracycline	*tet(Q)*	150 (90.4)	35 (87.5)	115 (91.3)	0.54
	tet(X2)	5 (3.0)	1 (2.5)	4 (3.2)	1.00
	tet(36)	3 (1.8)	0	3 (2.4)	1.00
	tet(X1)	1 (0.6)	0	1 (0.8)	1.00
Nitroimidazole	*nimJ*	2 (1.2)	0	2 (1.6)	1.00
	nimA	1 (0.6)	0	1 (0.8)	1.00
	nimE	1 (0.6)	0	1 (0.8)	1.00

Abbreviations: *cfiA*+ BF, *cfiA*-positive *Bacteroides fragilis*; *cfiA*− BF, *cfiA*-negative *Bacteroides fragilis*. [1] *p*-values were calculated by comparing the *cfiA*+ BF with the *cfiA*− BF groups.

Supplementary Table S5. Baseline demographics and clinical characteristics of patients with *B. fragilis* infections.

	Variable	All Patients (n = 166)	*cfiA*+ BF (n = 40)	*cfiA*− BF (n = 126)	p-Value [1]
		Number (Percent)			
Demographics	Median age (IQR)	73.0 (60.3–85.0)	72.5 (60.5–85.0)	73.5 (60.3–86.0)	0.67
	Age ≥ 65	109 (65.7)	25 (62.5)	84 (66.7)	0.63
	Male gender	78 (47.0)	18 (45.0)	60 (47.6)	0.77
	Residential care home	24 (14.5)	7 (17.5)	17 (13.5)	0.53
Charlson Comorbidity Index	Median score (IQR)	4.0 (2.0–6.8)	5.0 (3.0–6.0)	4.0 (2.0–7.0)	0.83
	0	25 (15.1)	6 (15.0)	19 (15.1)	
	1–2	18 (10.8)	3 (7.5)	15 (11.9)	
	3–4	42 (25.3)	10 (25.0)	32 (25.4)	
	≥ 5	81 (48.8)	21 (52.5)	60 (47.6)	
Comorbidities	Diabetes mellitus	59 (35.5)	20 (50.0)	39 (31.0)	0.03
	Active malignancy	35 (21.1)	10 (25.0)	25 (19.8)	0.49
	Chronic kidney disease	40 (24.1)	14 (35.0)	26 (20.6)	0.06
	Liver cirrhosis	1 (0.6)	0	1 (0.8)	1.00
	Heart disease	32 (19.3)	8 (20.0)	24 (19.0)	0.89
Medical history	Recent surgery	8 (4.8)	4 (10.0)	4 (3.2)	0.10
	Recent immunosuppressant use	10 (6.0)	2 (5.0)	8 (6.3)	1.00
Antibiotic exposure	Recent antibiotic use	51 (30.7)	14 (35.0)	37 (29.4)	0.50
	Carbapenem	7 (4.2)	2 (5.0)	5 (4.0)	0.68
	Beta-lactam/beta-lactamase inhibitor combinations	33 (19.9)	9 (22.5)	24 (19.0)	0.63
Presumed source of infection	Abdomen	64 (38.6)	15 (37.5)	49 (38.9)	0.88
	Skin and soft tissue	56 (33.7)	13 (32.5)	43 (34.1)	0.85
	Genital tract	16 (9.6)	5 (12.5)	11 (8.7)	0.54
	Septicemia with uncertain source	23 (13.9)	5 (12.5)	18 (14.3)	0.78
	Others	7 (4.2)	2 (5.00)	5 (4.0)	0.68
Clinical characteristics	Fever (> 37.8 °C) [2]	68/158 (43.0)	16/39 (41.0)	52/119 (43.7)	0.77

Abbreviations: *cfiA*+ BF, *cfiA*-positive *Bacteroides fragilis*; *cfiA*− BF, *cfiA*-negative *Bacteroides fragilis*; IQR, interquartile range. [1] p-values were calculated by comparing the *cfiA*+ BF with the *cfiA*− BF groups. [2] Eight patients (one *cfiA*+ BF and seven *cfiA*− BF) without temperature records were excluded.

Supplementary Table S6. Multivariate logistic regression for risk factors of *cfiA*-positive *B. fragilis* infections.

Variable	Odds Ratio (OR)	95% Confidence Interval (95% CI)	*p*-Value
Recent surgery	0.34	0.08–1.49	0.15
Diabetes mellitus	0.53	0.25–1.13	0.10
Chronic kidney disease	0.55	0.24–1.23	0.14

Supplementary Table S7. Treatment and clinical outcomes of patients with *B. fragilis* infections.

	Variable	All Patients (n = 166)	*cfiA*+ BF (n = 40)	*cfiA*− BF (n = 126)	p-Value [1]
		Number (Percent)			
Empirical antibiotics	Amoxicillin–clavulanic acid	93 (56.0)	19 (47.5)	74 (58.7)	0.21
	Piperacillin–tazobactam	29 (17.5)	6 (15.0)	23 (18.3)	0.64
	Cefoperazone–sulbactam	1 (0.6)	1 (2.5)	0	0.24
	Meropenem	8 (4.8)	3 (7.5)	5 (4.0)	0.40
	Ertapenem	4 (2.4)	1 (2.5)	3 (2.4)	1.00
	Metronidazole	10 (6.0)	5 (12.5)	5 (4.0)	0.06
	Clindamycin	1 (0.6)	1 (2.5)	0	0.24
	Absence of empirical antibiotics with expected activity against *B. fragilis*	25 (15.1)	6 (15.0)	19 (15.1)	0.99
	Appropriate empirical antibiotics [2]	87/165 (52.7)	17/39 (43.6)	70 (55.6)	0.19
Targeted antibiotics	Amoxicillin–clavulanic acid	60 (36.1)	12 (30.0)	48 (38.1)	0.35
	Piperacillin–tazobactam	37 (22.3)	9 (22.5)	28 (22.2)	0.97
	Cefoperazone–sulbactam	1 (0.6)	0	1 (0.79)	1.00
	Meropenem	25 (15.1)	10 (25.0)	15 (11.9)	0.04
	Ertapenem	9 (5.4)	1 (2.5)	8 (6.3)	0.69
	Metronidazole	53 (31.9)	11 (27.5)	42 (33.3)	0.49
	Clindamycin	4 (2.4)	1 (2.5)	3 (2.4)	1.00
	Absence of targeted antibiotics with expected activity against *B. fragilis*	14 (8.4)	5 (12.5)	9 (7.1)	0.33
	Appropriate targeted antibiotics	120 (72.3)	25 (62.5)	95 (75.4)	0.11
Interventions	Early source control	84 (50.6)	21 (52.5)	63 (50.0)	0.78
	ICU admission	13 (7.8)	5 (12.5)	8 (6.3)	0.31
Outcome	Duration of hospital stay ≥ 30 days [3]	48/157 (30.6)	15/37 (40.5)	33/120 (27.5)	0.13
All-cause mortality	7 days	7 (4.2)	3 (7.5)	4 (3.2)	0.36
	30 days	22 (13.3)	7 (17.5)	15 (11.9)	0.36
	90 days	40 (24.1)	10 (25.0)	30 (23.8)	0.88

Abbreviations: *cfiA*+ BF, *cfiA*-positive *Bacteroides fragilis*; *cfiA*− BF, *cfiA*-negative *Bacteroides fragilis*. [1] p-values were calculated by comparing the *cfiA*+ BF with the *cfiA*− groups. [2] One patient (*cfiA*+ BF) treated with cefoperazone–sulbactam only was excluded because the appropriateness of therapy could not be determined due to the absence of an EUCAST clinical breakpoint. [3] Nine outpatient patients (three *cfiA*+ BF and six *cfiA*− BF) were excluded.
